# Diagnostic accuracy of an artificial neural network compared with statistical quantitation of myocardial perfusion images: a Japanese multicenter study

**DOI:** 10.1007/s00259-017-3834-x

**Published:** 2017-09-26

**Authors:** Kenichi Nakajima, Takashi Kudo, Tomoaki Nakata, Keisuke Kiso, Tokuo Kasai, Yasuyo Taniguchi, Shinro Matsuo, Mitsuru Momose, Masayasu Nakagawa, Masayoshi Sarai, Satoshi Hida, Hirokazu Tanaka, Kunihiko Yokoyama, Koichi Okuda, Lars Edenbrandt

**Affiliations:** 1 Kanazawa University Hospital, Kanazawa, Japan; 2 Nagasaki University Hospital, Nagasaki, Japan; 3 Hakodate Goryoukaku Hospital, Hakodate, Japan; 4 National Cerebral and Cardiovascular Center, Suita, Japan; 5 Tokyo Medical University Hachioji Medical Center, Hachioji, Japan; 6 Hyogo Brain and Heart Center, Himeji, Japan; 7 Tokyo Women’s Medical University, Tokyo, Japan; 8 Akita City Hospital, Akita, Japan; 9 Fujita Health University Hospital, Toyoake, Japan; 10 Tokyo Medical University Hospital, Tokyo, Japan; 11 Tokyo Medical University Ibaraki Medical Center, Ibaraki, Japan; 12 Public Central Hospital of Matto Ishikawa, Hakusan, Japan; 13 Kanazawa Medical University, Uchinada, Kahoku, Japan; 14 University of Gothenburg, Gothenburg, Sweden

**Keywords:** Artificial intelligence, Diagnostic imaging, Coronary artery disease, Nuclear cardiology, Computer-aided diagnosis

## Abstract

**Purpose:**

Artificial neural networks (ANN) might help to diagnose coronary artery disease. This study aimed to determine whether the diagnostic accuracy of an ANN-based diagnostic system and conventional quantitation are comparable.

**Methods:**

The ANN was trained to classify potentially abnormal areas as true or false based on the nuclear cardiology expert interpretation of 1001 gated stress/rest ^99m^Tc-MIBI images at 12 hospitals. The diagnostic accuracy of the ANN was compared with 364 expert interpretations that served as the gold standard of abnormality for the validation study. Conventional summed stress/rest/difference scores (SSS/SRS/SDS) were calculated and compared with receiver operating characteristics (ROC) analysis.

**Results:**

The ANN generated a better area under the ROC curves (AUC) than SSS (0.92 vs. 0.82, *p* < 0.0001), indicating better identification of stress defects. The ANN also generated a better AUC than SDS (0.90 vs. 0.75, *p* < 0.0001) for stress-induced ischemia. The AUC for patients with old myocardial infarction based on rest defects was 0.97 (0.91 for SRS, *p* = 0.0061), and that for patients with and without a history of revascularization based on stress defects was 0.94 and 0.90 (*p* = 0.0055 and *p* < 0.0001 vs. SSS, respectively). The SSS/SRS/SDS steeply increased when ANN values (probability of abnormality) were >0.80.

**Conclusion:**

The ANN was diagnostically accurate in various clinical settings, including that of patients with previous myocardial infarction and coronary revascularization. The ANN could help to diagnose coronary artery disease.

## Introduction

Myocardial perfusion images have been interpreted based on an integrated understanding of myocardial perfusion distribution at stress and rest, and the difference between these two conditions is used to differentiate stress-induced ischemia and infarction. Although visual interpretation is always the first step, nuclear cardiology trainees need to become competent in reading single-photon emission computed tomography (SPECT) images [1, 2]. On the other hand, quantitative analysis has usually consisted of myocardial segmentation into walls and segments, and the extent and severity of perfusion defects are expressed in terms of the size of an abnormal area and defect scores [3, 4]. Most nuclear cardiology software has taken this type of quantitative approach, and it has aided the visual evaluation of images. One advantage of quantitative analysis is that it has led nuclear cardiologists and nuclear medicine physicians to standardize interpretations among physicians and hospitals [5]. Perfusion defects and ischemia are quantified not only for diagnosis, but for the prognostic evaluation of future cardiac events [6].

Artificial intelligence including artificial neural networks (ANN), deep learning and machine learning has recently been applied to nuclear cardiology [7–11]. Artificial intelligence learns how to appropriately interpret images based on a large number of SPECT images with definitive diagnoses based on nuclear cardiology expert interpretations as the teacher. The ANN approach includes an appropriate combination of features of abnormalities, and it differs from statistical approaches using regional count distribution, which is typically based on means and deviations. The ANN approach simulates how experts learn the art of interpretation. Therefore, stenosis of coronary arteries is not a truth or a standard; the integrated judgment of abnormalities by experts is the standard. Based on such repetitive learning processes, artificial intelligence could provide support for decision-making [12, 13].

The ANN system trained in Sweden using Swedish databases has been applied to Swedish and Japanese MPI SPECT studies related to an ANN diagnosis from myocardial perfusion images (MPI) [9, 10]. Although the diagnostic accuracy was comparable to that of defect scoring methods, additional clinical experience has revealed that slight abnormalities seem to be underestimated, and thus improvements in diagnostic accuracy are needed.

The purpose of this multicenter project was, therefore, to retrain the ANN system using a new Japanese MPI database and to develop a high-performance diagnostic system for coronary artery disease. The diagnostic ability of the ANN system was further compared with expert readings of another dataset from a multicenter validation database.

## Methods

### Patients for creating a training database

Data from 1001 patients who underwent stress/rest MPI were accumulated from 12 institutions in Japan. The entry criteria included confirmed and suspected coronary artery disease, and a confirmed diagnosis of the presence or absence of myocardial ischemia and/or infarction. The exclusion criteria comprised patients aged <20 years, arrhythmia causing inappropriate electrocardiographic gating, left bundle branch block (to avoid false positive defects), idiopathic and other types of severe cardiomyopathy, as well as moderate or severe valvular heart diseases. Patient data included age, sex, height, weight, risk factors, results of coronary angiography or coronary CT angiography (CCTA), and a history of percutaneous coronary intervention (PCI) or coronary artery bypass grafting (CABG). We tried to include either stress-induced ischemia or infarction in at least 50% of the patients when the MPI data were collected to train the ANN system.

### Patients for validation study

After the initial creation of the database, additional patients were registered using criteria similar to that of the training database. Data from 364 patients collected from nine hospitals served as the validation dataset.

### Myocardial perfusion imaging

Datasets were derived from all patients using exercise or adenosine stress MPI with electrocardiographic gating and non-gating (Table 1). ^99m^Tc-labeled hexakis-2-methoxyisobutylisonitrile (MIBI) was used in all participating institutions, and the second injection dose was 2- to 3-fold higher than the first, resulting in a total administered dose of 740 to 1110 MBq. All institutional protocols for stress and image acquisition followed standard exercise and adenosine stress MPI protocols [1, 14]. All image data were anonymized in the "Digital Imaging and Communication in Medicine" (DICOM) format at each institution and uploaded into a custom-designed server. The only patient information in the image database in the server was serial number, age and sex.

**Table 1:** Patient demographics in the training and validation databases

Items	Training database: value, %, means ± SD (range)	Validation database: value, %, means ± SD (range)	p
Number of participants	1001	364	–
Age (years)	69 ± 10	71 ± 10	0.0011
Male (%)	75%	73%	0.48
Height (male, cm)	165 ± 7	165 ± 7	0.98
Weight (male, kg)	66 ± 12	67 ± 11	0.48
Body mass index (male, kg/m^2^)	24 ± 4	24 ± 3	0.64
Height (female, cm)	151 ± 7	151 ± 6	0.12
Weight (female, kg)	54 ± 11	55 ± 10	0.18
Body mass index (female, kg/m^2^)	24 ± 4	24 ± 4	0.46
Pharmacological stress (%)	70%	82%	0.0001
Number of vessel stenosis ≥75% (1-, 2- and 3-vessel disease)	391 (156, 123, 112)	225 (78, 82, 65)	0.0001
Hypertension (%)	73%	75%	0.40
Diabetes mellitus (%)	47%	39%	0.019
Dyslipidemia (%)	65%	66%	0.79
History of myocardial infarction (%)	27%	31%	0.17
History of PCI (%)	38%	39%	0.87
History of CABG (%)	4%	3%	0.53
ANN stress defect	–	0.63 ± 0.37	–
Presence of stress defect (%)	71%	73%	0.54
ANN ischemia	–	0.51 ± 0.34	–
Presence of ischemia (%)	59%	59%	1.00
ANN rest defect	–	0.54 ± 0.38	–
Presence of rest defect (%)	57%	56%	0.76
Summed stress score	9.5 ± 9.9 (0–53)	9.5 ± 9.8 (0–52)	1.00
Summed rest score	7.0 ± 8.6 (0–45)	7.1 ± 8.8 (0–49)	0.85
Summed difference score	3.3 ± 3.9 (0–26)	3.1 ± 3.3 (0–21)	0.38
Rest end-diastolic volume (mL)	105 ± 38 (38–325)	107 ± 46 (34–341)	0.42
Rest end-systolic volume (mL)	38 ± 29 (5–250)	37 ± 29 (3–246)	0.57
Rest ejection fraction (%)	67 ± 13 (19–97)	63 ± 14 (25–92)	0.0001

Abbreviations: ANN, artificial neural network (probability of abnormality in this table); CABG, coronary artery bypass grafting; PCI, percutaneous coronary intervention

### Training the artificial neural network

The left ventricle (LV) was segmented using a three-dimensional heart-shaped active-shape model reconstructed with short-axis slice images [15, 16]. A specific software algorithm was used for this analysis (cardioREPO software, FUJIFILM RI Pharma Co. Ltd., Tokyo, Japan; EXINI Diagnostics AB, Lund, Sweden). Areas of possible perfusion abnormalities in stress and rest images (stress and rest defects, respectively) were segmented using a method that mimicked analyses by physicians who visually interpret defects. Candidate regions with abnormalities were identified using deformable models of circular topology based on an active contour framework, and fit to the image based on edge and intensity information [17]. Stress images were subtracted from rest images to identify stress-induced ischemia. Appropriate features were extracted using the characteristics of shape, extent, location, count, perfusion homogeneity, regional motion, wall thickening and sex. The best combination of features was determined for stress, rest and difference datasets, and these features served as inputs for the ANN analysis.

Nuclear cardiology experts at each institution judged all regions in MPI data as abnormal (true) or normal (false). The software algorithm identified 5685 candidate regions, but whether or not an abnormality is true was determined by nuclear cardiologists and nuclear physicians. The interpreters used original myocardial stress and rest images and polar maps, but not quantitative data, such as defect scores for detecting abnormalities. After all data were accumulated at the core center, six nuclear cardiology experts reconfirmed the appropriateness of the judgment without clinical information. Two experts interpreted the data during this process and, if necessary, modified the judgment with referral to other expert opinions to reach consensus. The final judgments of the candidate regions made at the core center served as the gold standard with which to train the ANN system.

Based on the initial ANN training, discordance between visual and ANN findings were again selected and used for appropriately adjusting diagnostic thresholds. In the final output of the software, the ANN value indicated the probability of an abnormality under conditions of 0.0 (definitely normal), 0.5 (borderline) and 1.0 (definitely abnormal) for stress defects, rest defects and induced ischemia.

### Processing by the artificial neural network in the software package

Four DICOM stress/rest gated and ungated images were transferred from the nuclear medicine dedicated computer system to an on-line connected (off-line acceptable) Windows computer with the aid of a custom-made launcher. After selecting a patient dataset, all processing steps including LV segmentation, polar map generation, detection of abnormal regions, judgment of the abnormality with the ANN system, scoring based on Japanese Society of Nuclear Medicine (JSNM) working group normal databases [18, 19], LV functional analysis including wall motion, volume, ejection fraction and diastolic function, and phase dyssynchrony analysis were performed within 10 s (64-bit operating system, Windows 10) [10, 16, 19–21]. This processing was automatic without operator interaction, but LV contour detection can be modified if required. The ANN system displays a list of regions including the location, extent, severity and ischemia/infarct with the probability of an abnormality (ANN value) in each region.

### Defect scoring

Myocardial perfusion defects were scored using a standard 17-segment model, and abnormalities were defined as normal, slightly, moderately and severely decreased, and defective (scores 0, 1, 2, 3, and 4, respectively) in each segment [3, 4]. Scores were validated in automated analysis using cardioREPO software, and the correlation was good compared with that determined using QPS (Cedars Sinai Medical Center, Los Angeles, CA, USA) [10]. The maximum score was 68 points for summed stress/rest/difference scores (SSS/SRS/SDS). Normal MPI databases were constructed according to the JSNM working group database that includes stress/rest normal SPECT data with non-attenuation-corrected Anger camera images [18, 19].

### Statistics

Data are shown as means ± standard deviation (SD). Differences between groups were assessed using the one-way analysis of variance, Student's *t* tests and F tests. A contingency table was analyzed with Fisher exact tests. Receiver operating characteristics (ROC) were analyzed, and the area under the curve (AUC) was calculated. The cutoff was determined using either an ANN value (probability of abnormality) of 0.50 or the highest value of (sensitivity + specificity − 1) depending on the analysis. The statistics software was JMP version 12 (SAS Institute Inc., Cary, NC, USA), and Mathematica 12 (Wolfram Research Inc., Champaign, IL, USA) was also used for some mathematical calculations. A *p* value <0.05 was considered significant.

## Results

### Training database

Table 1 summarizes the characteristic of the training databases that included stress defects (71%), rest defects (57%) and ischemia (59%). Old myocardial infarction (OMI) and revascularization were found in 27% and 42% of the patients, respectively. The AUC of the training database calculated to obtain results concordant with those of the experts were 0.912 (sensitivity 86%, specificity 77%, accuracy 82%) for stress defects, 0.834 (sensitivity 83%, specificity 74%, accuracy 78%) for rest defects and 0.888 (sensitivity 82%, specificity 77%, accuracy 80%) for ischemia.

### Validation database

Background conditions and associated diseases did not significantly differ between the validation and the training databases for most of the variables (Table 1). Rates of stress defects, rest defects and ischemia were similar. However, the validation database had a higher incidence of pharmacological stress and of coronary stenosis, and a lower ejection fraction than the training database.

### Interpretation of the ANN system

**Fig. 1:**
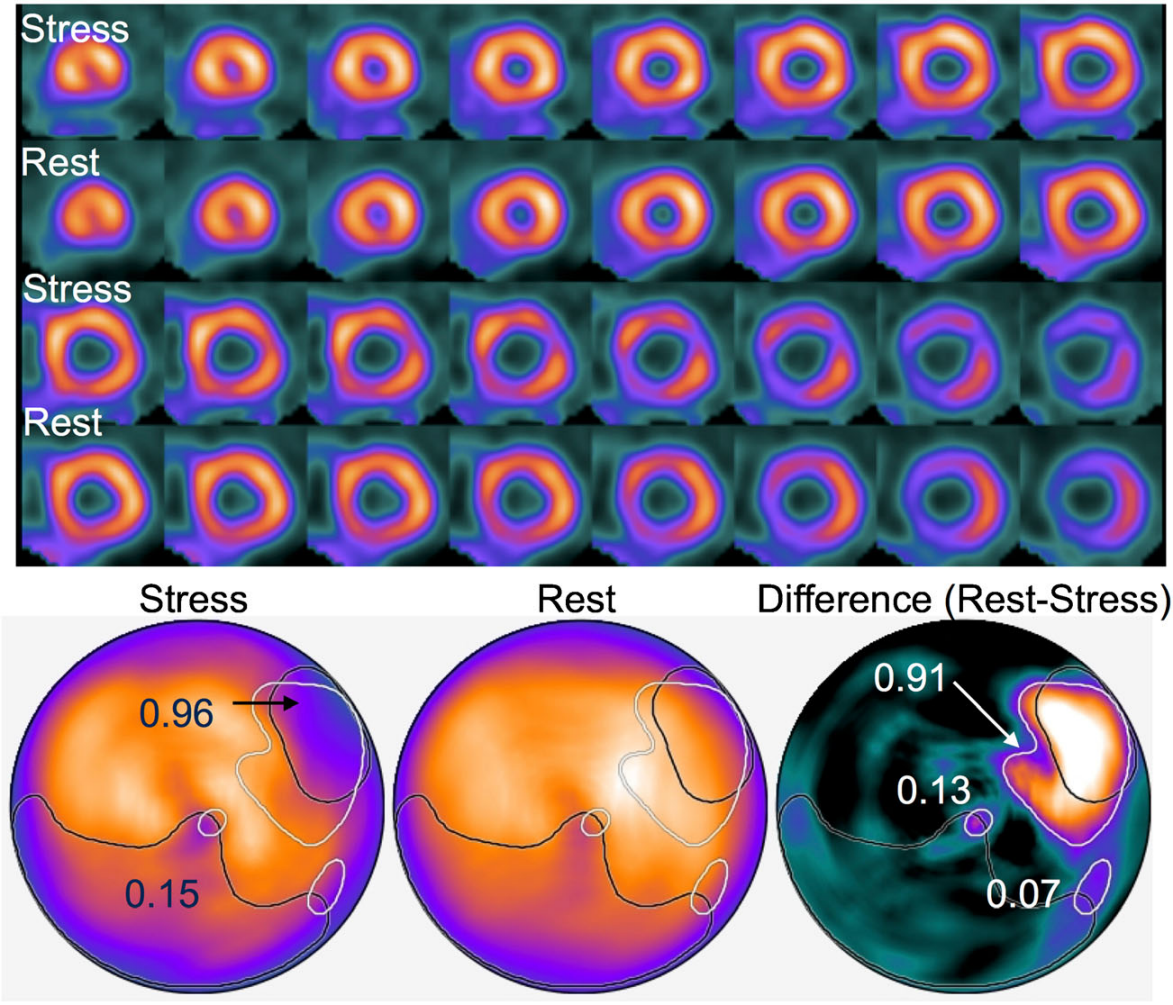
Myocardial perfusion study and artificial neural network (ANN) analysis of 70-year-old man after percutaneous coronary intervention to the left circumflex coronary artery. Numbers indicate probability of abnormality. Basal lateral ischemia is evident in short-axis images (upper panel), whereas the ANN system identified abnormality in stress (probability) and subtraction (probability) images with probabilities of 0.96 and 0.91, respectively. Other regions with probability of <0.5 were considered insignificant

Figure 1 shows the standard output generated using the ANN. This patient had 99% stenosis of the circumflex coronary artery (#11). Six months after stenting he developed chest pain, which was assessed by MPI. The ANN judged the high lateral region as abnormal in both stress and subtraction images with high probability.

### ROC analysis for scores and the ANN

**Fig. 2:**
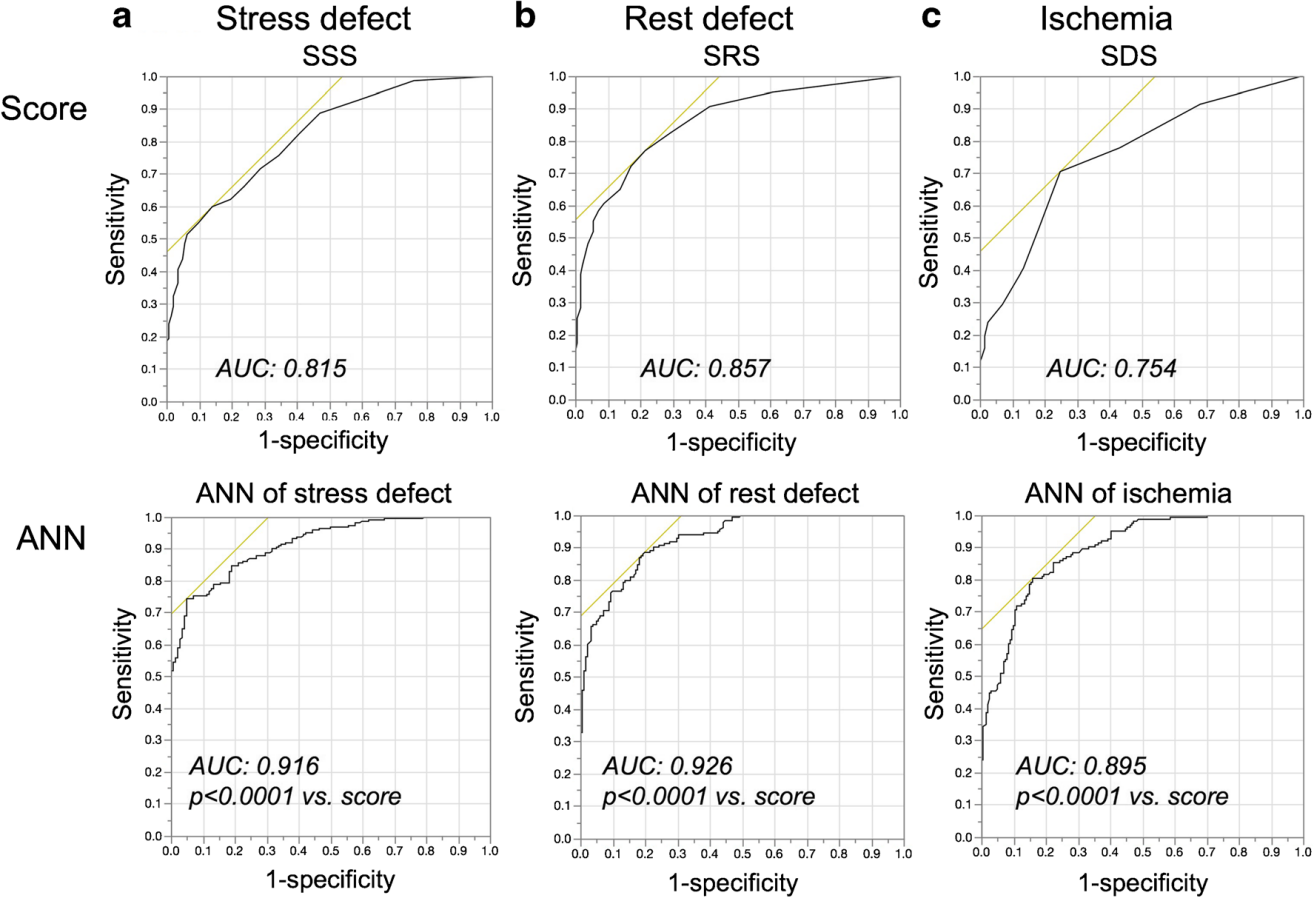
Receiver operating characteristics (ROC) analysis of stress defect (**a**), rest defect (**b**) and ischemia (**c**) using the scoring method (upper panel) and the artificial neural network (ANN; lower panel). All areas under ROC curves (AUC) were higher for the ANN (*p* < 0.0001)

The AUC values of the stress defect were 0.815 and 0.916 (χ^2^ = 34.4, *p* < 0.0001) for the scoring system and the ANN, respectively (Fig. 2). Similarly, the AUC was 0.857 vs. 0.926 (χ^2^ = 21.9, p < 0.0001) for rest defects, and 0.754 vs. 0.895 (χ^2^ = 34.2, *p* < 0.0001) for ischemia, respectively, indicating a higher AUC for the ANN compared with the scoring method.

**Fig. 3:**
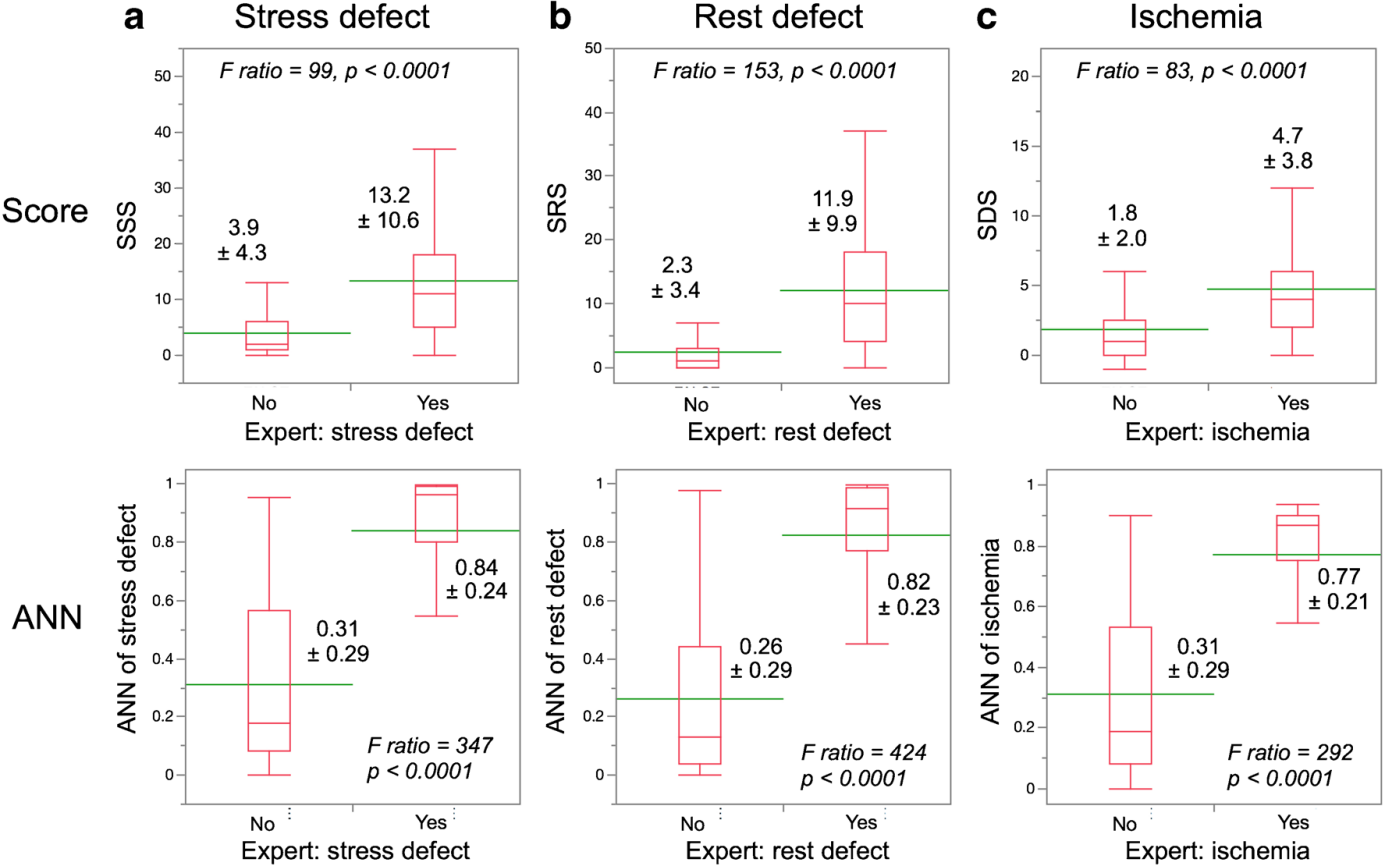
Comparison of the scoring method (upper panel) and artificial neural network (ANN; lower panel) based on expert judgments. Positive and negative judgments significantly differed in all comparisons of stress defects (**a**), rest defects (**b**) and ischemia (**c**)

Average scores for stress defects were 13.2 ± 10.6 vs. 3.9 ± 4.3 (*p* < 0.0001, F ratio = 99) in patients with and without stress defects, respectively (Fig. 3). The values for stress defects determined using the ANN in patients with and without stress defects were 0.84 ± 0.24 vs. 0.31 ± 0.29 (*p* < 0.0001, F ratio = 347). Similarly, although both scores and ANN values under conditions of rest, stress and ischemia significantly differed between positive and negative expert readings (*p* < 0.0001), the F ratios were higher when determined by the ANN than the scoring method.

### ROC analysis in subgroups of patients

The ability of the ANN to detect abnormalities in patient subgroups with and without OMI or revascularization was generally good, with an AUC of ≥0.89 (Table 2). The AUC values for the ANN to detect stress defects in patients with and without OMI were 0.976 and 0.892, respectively. In patients with OMI, the AUC values to detect rest defects using SRS and the ANN were 0.905 and 0.974, respectively. The AUC values for detecting stress defects with and without a history of revascularization were 0.939 and 0.900, respectively. The AUC in all subgroups was higher for the ANN than the scoring method.

**Table 2:** Receiver operating characteristics (ROC) analysis of subgroups in the validation study

	ANN/score	AUC	Standard error	Lower 95%	Upper 95%	*p* vs. score
Stress defect with and without OMI						
Without OMI	ANN	0.892	0.020	0.846	0.925	<0.0001
	SSS	0.771	0.030	0.708	0.824	
With OMI	ANN	0.976	0.014	0.929	0.992	0.0036
	SSS	0.890	0.033	0.807	0.940	
Rest defect						
With OMI	ANN	0.974	0.014	0.925	0.991	0.0061
	SRS	0.905	0.029	0.831	0.949	
Stress defect with and without history of revascularization						
No revascularization	ANN	0.900	0.020	0.854	0.932	<0.0001
	SSS	0.781	0.030	0.716	0.835	
Revascularization	ANN	0.939	0.019	0.889	0.967	0.0055
	SSS	0.863	0.031	0.790	0.913	
Ischemia with and without history of revascularization						
No revascularization	ANN	0.898	0.020	0.853	0.931	<0.0001
	SDS	0.771	0.032	0.703	0.827	
Revascularization	ANN	0.889	0.027	0.823	0.932	0.0002
	SDS	0.727	0.042	0.636	0.802	

Abbreviations: ANN, artificial neural network; AUC, area under the curve; OMI, old myocardial infarction; ROC, receiver-operating characteristics; SDS, summed difference score; SRS, summed rest score; SSS, summed stress score

According to ≥75% of coronary stenosis, we divided the patients into groups according to their having 0–1-, or 2–3-vessel disease. The AUC values were 0.895 and 0.785 when determined using the ANN and SSS, respectively (χ^2^ = 24.2, *p* < 0.0001) for patients with 0–1-vessel disease and 0.945 and 0.838, respectively, for patients with 2–3-vessel disease (χ^2^ = 11.0, *p* = 0.0009), indicating that the ANN had better diagnostic accuracy.

### Relationship between scores and ANN values

**Fig. 4:**
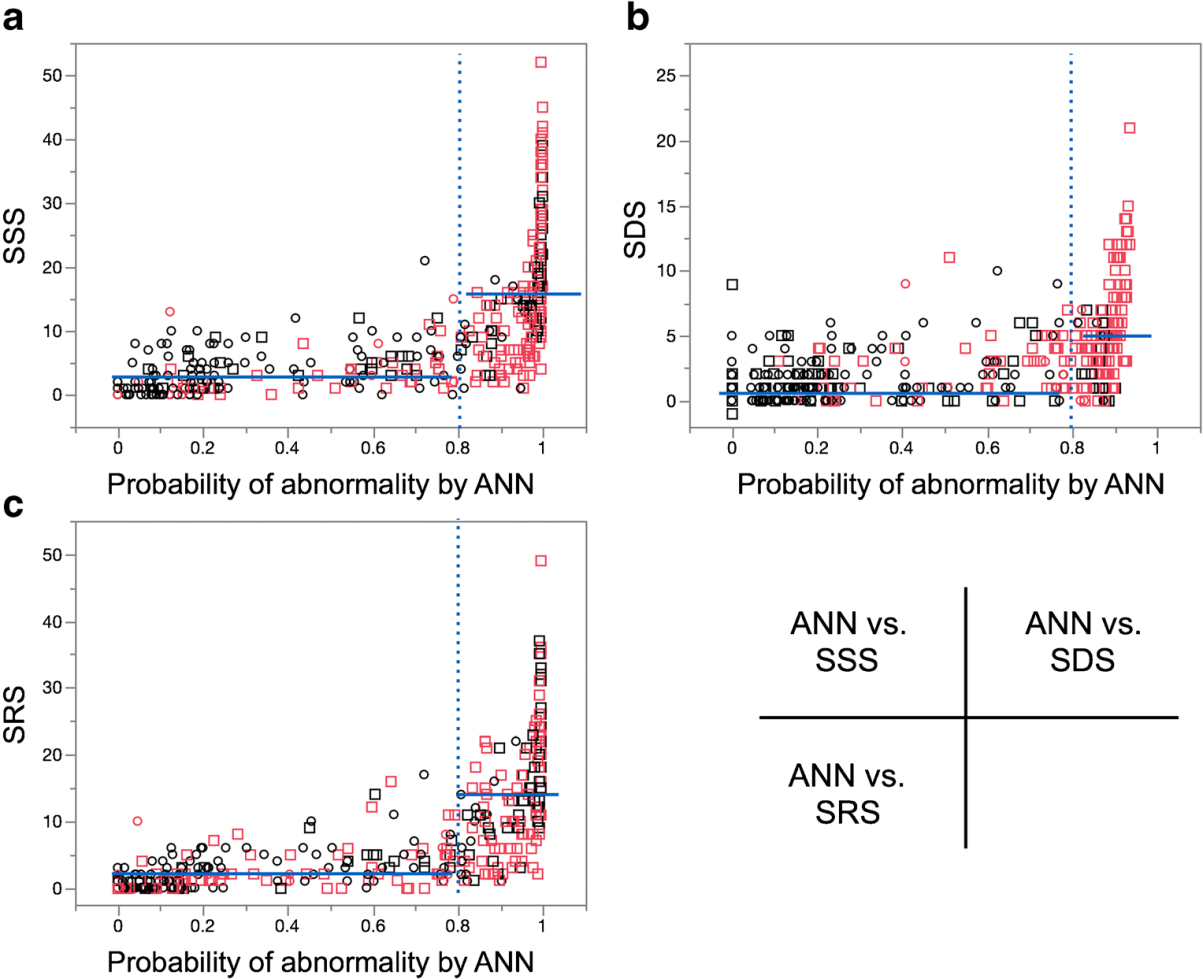
Relationship between scoring methods and probability of abnormality judged by the ANN. Dotted vertical lines indicate probability of 0.8, and blue lines indicate mean values for probabilities of <0.8 and ≥0.8. Squares and circles denote positive and negative stress defect, respectively by expert interpretations. Red and black marks denote positive and negative ischemia, respectively by expert interpretations

Figure 4 shows a non-linear relationship between scores and the ANN values in the validation group. Although the threshold of abnormality using the ANN was a probability of 0.5, the scores steeply increased when the ANN probability was ≥0.8. The SSS was 3.4 ± 3.5 in the range of ANN probability <0.80, but 15.8 ± 10.3 when in the probability range of ≥0.80 (*p* < 0.0001) determined by the ANN. The SDS values were 1.8 ± 4.7 and 4.7 ± 3.8 when the ANN probability was <0.8 and ≥0.8, respectively, and the SRS values were 2.3 ± 3.0 and 14.2 ± 9.8 when the ANN probability was <0.8 and ≥0.8, respectively (*p* < 0.0001).

### Diagnostic accuracy of the ANN compared with expert interpretation

Diagnostic accuracy using criteria of significant coronary stenosis of ≥75% with either coronary angiography or CCTA was examined in a subgroup of patients (*n* = 220), excluding those after coronary revascularization. Using these criteria, 0, 1-, 2- and 3-vessel disease was found in 98 (45%), 44 (20%), 41 (19%) and 37 (17%) patients, respectively. The AUC values using stress defects for detecting 1-, 2- and 3-vessel disease were 0.69, 0.67 and 0.69 by expert interpretation, and those by the ANN were 0.68, 0.66 and 0.72, respectively, showing an overall AUC of 0.66 (95% CI: 0.60–0.72) by expert interpretation and 0.65 (0.58–0.72) by ANN (*p* = 0.74) for detecting at least one coronary stenosis.

## Discussion

This multicenter study aimed to develop an ANN-based diagnosis for myocardial perfusion stress/rest gated and ungated SPECT images. The diagnostic ability of the ANN became comparable to that of nuclear cardiology expert interpretation and was better than conventional semi-quantitative defect scoring. Concordance was reasonable between experts and the ANN even for subgroups of patients with infarction and revascularization because the nuclear cardiologist interpretation taught the ANN.

### Comparison with conventional methods

Basic approaches of artificial intelligence-based diagnostic systems differ from the diagnostic steps based on conventional statistical methods [13]. Normal or standard databases are created from individuals with a low likelihood of coronary artery disease for statistical analysis, and the limits of normal distribution are determined for all myocardial data points. Combinations of the average and the deviation or ratio (%) of threshold counts are used to measure degrees of abnormalities, which are expressed as defect scores. Such statistical analysis provides consistent diagnosis using common databases fitted for populations [22]. It also serves as an aid to clinical diagnosis and has, therefore, been used in most quantitation software packages [5, 23–25]. Quantitative diagnosis using a scoring method is convenient for measuring amounts of ischemia, and for guiding appropriate indications for coronary revascularization [6].

Here, we scored defects using a JSNM working group database of myocardial SPECT images for this purpose [18, 19], which was compared with ANN-based diagnosis. On the other hand, the ANN uses a completely different approach [9, 10, 12]. The software in this study first identified candidate areas of abnormality from >5000 candidate regions based on a specific algorithm, and learned the expert interpretation. Based on the notion that the expert diagnosis is true, the most appropriate features were determined for stress, rest and difference images. In the clinical setting, MPI diagnosis is not simply based on average counts on polar maps, but on the integration of myocardial counts, size and shape of defects, wall motion and systolic thickening, gender and other parameters. Although experts might have unintentionally taken these factors into consideration, the ANN was capable of learning this comprehensive diagnostic process.

Artificial intelligence such as a support vector machine (SVM) and machine learning technologies has generated promising results for the diagnosis and prognosis of patients with coronary artery disease. The AUC for SVM (0.92) is significantly better than total perfusion deficit (0.90) when the SVM algorithm is applied, and its diagnostic accuracy is comparable to the overall accuracy of visual readers [8]. Machine learning that included a combination of clinical and CCTA data predicted 5-year all-cause mortality more effectively than existing clinical or CCTA metrics alone [11]. Since artificial intelligence considers a greater number and complexity of variables than humans, it might be applied for wider indications in the near future.

### The meaning of true abnormality

Since the purpose of this project was to create an ANN with diagnostic ability that was similar to that of experts, true abnormality was defined by consensus among expert interpretations. Popular methods for evaluating diagnostic accuracy to date have used coronary stenosis as the gold standard by which sensitivity and specificity are calculated. However, all three coronary territories might not be judged as abnormal in patients with three-vessel disease. If the most severe stenosis is a culprit lesion and only one territory appears abnormal, even experts might judge only one coronary territory as abnormal. Moreover, some patients who have been diagnosed with myocardial infarction might have recovered perfusion to some extent. Others might have insufficient coronary blood flow supply even from the revascularized coronary artery. Recent indications for MPI include not only patients who are untreated, but also those with many complex pathophysiological conditions such as myocardial ischemia, fibrosis and modifications after revascularization and medical therapies. In fact, 30% of patients included in the present study had OMI and 40% had a history of revascularization. Although we did not use coronary stenosis as the gold standard, the diagnostic ability of the ANN was significantly better than scoring, and comparable to that of experts, indicating a good foundation for further clinical application.

However, even when coronary stenosis of ≥75% was used as a diagnostic criterion, the diagnostic accuracy using ROC analysis was comparable between the expert interpretation and ANN. The reason for excluding patients with history of coronary revascularization was that “truth” regarding the presence of remaining ischemia or defects was not confirmed in these patients. Although this analysis was not the goal of the present study, strict criteria of ischemia using coronary stenosis and fractional flow reserve could be evaluated in further studies.

### Training and validation databases

The interpretation of myocardial perfusion images during ANN training could affect final diagnostic accuracy. The diagnostic accuracy of the ANN in a previous study of a Swedish database was 92% for abnormalities at stress and 87% for ischemia [9]. In addition, the same ANN generated an AUC of ≥0.88 in a Japanese population [10]. After accumulating clinical experience with this software, we found that a minor degree of ischemia was overlooked in a Japanese population. We therefore tried to judge even a slight degree of abnormality as abnormal, which is in agreement with our clinical practice. The ANN learned this tendency of expert interpretation during this project. However, the probability of an abnormality, namely the calculated ANN value in regions with a minor degree of ischemia, might be somewhat lower than that of a definite perfusion defect or ischemia. Overall, since a common stress/rest protocol using ^99m^Tc-MIBI was applied, we suppose that the software can be applied to routine clinical investigations at many hospitals. The applicability of the ANN-based system to diagnostic ^201^Tl and other SPECT studies using equipment such as cadmium-zinc-telluride detectors should be separately investigated.

### Value of ANN and scoring methods

The non-linear relationship between ANN values and defect scoring was a notable feature that seems in agreement with human decision-making processes. Nuclear cardiologists do not usually judge findings with defect scores <4 as abnormal, but will definitely do so when scores are ≥8. Thus, the likelihood of an abnormal probability steeply increasing between 4 and 8 is understandable. Considering this non-linear relationship, the scoring method cannot be replaced by the ANN. Although 10% ischemia of the left ventricle is used as a guide to coronary revascularization [6, 26], the ANN does not provide a comparable threshold of ischemic severity. However, the ANN can emphasize a possible abnormal region with a degree of probability, which can suggest a diagnosis. Avoidance of overlooking positive findings and over-diagnosing minor abnormalities might be helpful for nuclear cardiology trainees. Since cardiologists and radiologists are not always specialists in nuclear cardiology, an ANN-based suggestion might help enhance confidence in a diagnosis. Even some nuclear cardiology specialists tend to interpret findings with high sensitivity (active reading for abnormality), and others with high specificity (modest reading). The average reading provided by the ANN could thus be a second opinion for such specialists.

Lastly, all perfusion processing, defect scoring and functional analysis are automatically calculated by the software within 10 s. Therefore, if the data transfer protocol from a nuclear-dedicated computer to Windows PC and connection to an institutional picture archiving and communication system (PACS) are appropriately constructed, the ANN software system could function in any institution.

### Limitation

The territories of the three coronary arteries were not included in the training process. Although the coronary territory and polar maps might roughly correspond, such as the anteroseptal region to the left anterior descending coronary artery, strict correspondence in border zones of coronary territories is difficult to determine. Although we could not determine coronary-based accuracy in this study due to its algorithm, fusion images with CCTA and perfusion maps with their probability of ischemia will effectively integrate information from stenosis and perfusion. As discussed above, we could not generate a severity score indicating 10% ischemia. To develop an ANN that can assess ischemic severity comparable to that of severity scoring, new large-scale training will be required. The judgment suggested by the ANN system was based on average readings of the MPI studies at 12 institutions. The variety of conditions is a limitation from the viewpoint of specific readings at these institutions, but this could conversely provide the advantage of averaged interpretation.

### Conclusion

Based on a new multicenter database trained by nuclear cardiology specialists, the diagnostic ability of the ANN was good with an AUC of >0.9 overall, including subgroups with and without MI and coronary revascularization. Further studies of large patient cohorts are indicated based on these promising outcomes.
